# The Association Between Abnormal Electrocardiogram Findings and the Ankle Brachial Index

**DOI:** 10.3390/medicina62061083

**Published:** 2026-06-02

**Authors:** Ben Li, Adam M. Khalil, Abdallah Bakeer, Abdelrahman Zamzam, Rawand Abdin, Mohammad Qadura

**Affiliations:** 1 Division of Vascular Surgery, St. Michael’s Hospital, Unity Health Toronto, University of Toronto, Toronto, ON M5B 1W8, Canada; benx.li@mail.utoronto.ca (B.L.);; 2 Department of Surgery, University of Toronto, Toronto, ON M5S 1A1, Canada; 3 Temerty Centre for Artificial Intelligence Research and Education in Medicine (T-CAIREM), University of Toronto, Toronto, ON M5S 1A1, Canada; 4 Institute of Medical Science, University of Toronto, Toronto, ON M5S 1A1, Canada; 5 Heart, Vascular, & Thoracic Institute, Cleveland Clinic Abu Dhabi, Abu Dhabi 112412, United Arab Emirates; 6 Department of Medicine, McMaster University, Hamilton, ON L8S 4L8, Canada; 7 Li Ka Shing Knowledge Institute, St. Michael’s Hospital, Unity Health Toronto, University of Toronto, Toronto, ON M5B 1W8, Canada

**Keywords:** electrocardiogram, ankle brachial index, peripheral artery disease, abnormal

## Abstract

*Background and Objectives*: Peripheral artery disease (PAD) is a common manifestation of systemic atherosclerosis associated with significant morbidity and mortality, yet it remains underdiagnosed due to limited routine screening and its often-asymptomatic presentation. Although the ankle–brachial index (ABI) is the gold standard diagnostic tool for PAD, its use may be limited in some settings because it requires specialized equipment and trained personnel. In contrast, the electrocardiogram (ECG) is widely available and routinely performed. Given that ECG abnormalities may reflect systemic cardiovascular disease, we investigated the association between ECG findings and ABI classification. *Materials and Methods*: This retrospective case–control study analyzed patients with and without PAD who received care from outpatient vascular clinics between March 2018 and February 2022. PAD was defined as ABI ≤ 0.9 or toe–brachial index ≤ 0.67 with abnormal pedal pulses. The most recent ECG performed within one year of ABI measurement was retrieved for each patient and classified as normal, borderline, or abnormal according to standardized guideline recommendations. The association between ECG category and ABI classification was assessed using chi-square testing and multivariable logistic regression adjusted for age and sex, with results reported as odds ratios (ORs) and 95% confidence intervals (CIs). Discriminatory performance of the ability of ECG findings to predict ABI classification was evaluated using the area under the receiver operating characteristic curve (AUROC) with 95% CI. Model calibration was assessed using the Hosmer–Lemeshow test. *Results*: Overall, 491 patients had paired ECG and ABI data. ECGs were categorized as abnormal (n = 345), borderline (n = 58), or normal (n = 88). The prevalence of abnormal ABI (≤0.9) was highest among patients with abnormal ECGs (45.8%), compared to borderline (34.5%) and normal ECGs (28.4%) (*p* = 0.0067). On multivariable logistic regression analysis adjusted for age and sex, abnormal ECG findings were associated with increased odds of abnormal ABI compared to normal ECGs (adjusted OR 2.07, 95% CI 1.24–3.46, *p* = 0.005), whereas borderline ECGs were not (OR 1.31, 95% CI 0.64–2.68, *p* = 0.455). ECG categorization demonstrated moderate discrimination (AUROC 0.73, 95% CI 0.68–0.78) and good calibration (Hosmer–Lemeshow χ^2^ 5.0, *p* = 0.76) for predicting abnormal ABI. *Conclusions*: In this retrospective case–control study, we found an association between abnormal ECG findings and abnormal ABI. These results support the concept that clinically significant ECG abnormalities may reflect systemic atherosclerotic burden rather than isolated cardiac pathology. Given the widespread availability and low cost of ECG testing, ECG interpretation may help identify patients who warrant further investigations, including PAD screening, vascular assessment, and risk-stratification, particularly in lower-resource settings without routine access to ABI testing. Prospective, multicenter studies are needed to validate these findings.

## 1. Introduction

Peripheral artery disease (PAD) affects over 200 million people worldwide [1,2]. It is a common manifestation of systemic atherosclerosis and is associated with substantial morbidity, mortality, and cardiovascular risk [1,2]. Despite its clinical significance, PAD remains underdiagnosed [3]. This is in part due to its frequently asymptomatic presentation and the limited use of screening tools in routine practice [3].

The ankle–brachial index (ABI) is a simple, non-invasive, and well-validated measure for the diagnosis of PAD, with an ABI ≤ 0.9 demonstrating high specificity for lower extremity arterial disease [4]. However, ABI measurement requires specialized equipment and trained personnel, which may limit its widespread implementation in certain settings, including low-resource and/or rural areas [4,5].

The electrocardiogram (ECG), by contrast, is a widely available, low-cost, and routinely performed diagnostic test in diverse patient populations across a variety of settings [6]. Globally, more than 300 million ECG tests are performed annually [7]. Abnormal ECG findings, including arrhythmias, conduction disturbances, and ischemic changes, often reflect underlying cardiovascular pathology and may serve as markers of systemic vascular disease [6]. Given that PAD and coronary artery disease (CAD) share common pathophysiological mechanisms rooted in atherosclerosis, we hypothesized that abnormalities detected on ECG may be associated with impaired peripheral perfusion as reflected by a reduced ABI [8].

Prior studies have demonstrated associations between ECG abnormalities and adverse cardiovascular outcomes; however, the relationship between standardized ECG interpretation and ABI classification has not been well characterized [9,10,11]. Most existing studies have focused on isolated ECG abnormalities, such as ST–T wave changes or conduction disturbances, rather than global ECG interpretation based on standardized guideline criteria [9,10,11]. As a result, it remains unclear whether routinely reported ECG categorizations of normal, borderline, or abnormal reflect underlying peripheral vascular disease burden or are associated with abnormal ABI findings [12]. In particular, the utility of categorizing ECGs as normal, borderline, or abnormal based on established guideline definitions remains unclear in the context of PAD detection and risk stratification [12].

Accordingly, the objective of this study was to evaluate the association between standardized ECG categorization and ABI classification in patients with and without PAD. We further sought to assess whether abnormal ECG findings were associated with increased odds of abnormal ABI and to evaluate the discrimination and calibration of ECG categorization for identifying patients with PAD.

## 2. Materials and Methods

### 2.1. Ethical Approval

Ethics approval for this study was granted by the Institutional Review Board of Cleveland Clinic Abu Dhabi on 1 March 2023 (IRB # A-2023-033). Informed consent for participation was not required as per local legislation by the Institutional Review Board of Cleveland Clinic Abu Dhabi, given that this was a retrospective analysis of anonymized data. The study procedures adhered to the ethical standards outlined in the Declaration of Helsinki [13].

### 2.2. Study Design

This retrospective case–control study was conducted and reported in accordance with the Strengthening the Reporting of Observational Studies in Epidemiology (STROBE) guidelines [14].

### 2.3. Patient Recruitment

Between March 2018 and February 2022, consecutive patients with and without PAD who received care from outpatient vascular clinics at our hospital were retrospectively analyzed. PAD was defined as an ABI ≤ 0.9 or a toe brachial index (TBI) ≤ 0.67, together with absent or diminished pedal pulses [15]. Non-PAD was defined as an ABI > 0.9 and a TBI > 0.67, with normal pedal pulses [15]. Exclusion criteria included acute limb ischemia, acute coronary syndrome, or elevated troponin within the preceding three months. Patients without an ECG performed within 1 year prior to their ABI measurement, and individuals without complete ECG interpretation and ABI classification data were excluded.

### 2.4. Electrocardiogram Interpretation

The most recent ECG performed within 1 year prior to the ABI measurement was used for analysis. Resting 12-lead ECGs were obtained using standardized acquisition protocols by trained clinical professionals, with patients in the supine position following a brief rest period, and electrode placement in accordance with clinical guidelines [12]. ECG tracings were independently interpreted by a single board-certified cardiologist who was blinded to ABI results and followed standardized clinical reporting practices [12]. ECG findings were categorized as abnormal, borderline, or normal based on joint recommendations by the American Heart Association, American College of Cardiology Foundation, and Heart Rhythm Society for standardized ECG interpretation [12]. A normal ECG was defined as sinus rhythm with normal rate and axis, normal intervals (PR, QRS, and QTc), and no evidence of chamber enlargement, conduction abnormalities, or ischemic ST–T wave changes [12]. A borderline ECG was defined as minor or nonspecific abnormalities that do not meet criteria for definite pathology, including borderline interval prolongation (e.g., PR 200–220 ms, QTc at upper limits of normal), mild axis deviation, low QRS voltage, or nonspecific ST–T wave changes without diagnostic criteria for ischemia [12]. An abnormal ECG was defined as definite clinically significant abnormalities, including arrhythmias (e.g., atrial fibrillation/flutter), conduction disturbances (e.g., bundle branch block, and second- or third-degree atrioventricular block), evidence of prior or acute myocardial infarction (pathologic Q waves, significant ST-segment elevation or depression), left or right ventricular hypertrophy with repolarization abnormalities, or other major findings requiring clinical evaluation [12].

### 2.5. ABI Measurement and Interpretation

ABI’s were measured in a certified vascular laboratory by dividing the higher of the dorsalis pedis (DP) or posterior tibial (PT) artery systolic pressures in each leg by the higher brachial systolic pressure [4]. Specifically, simultaneous and automated measurements with validated multi-cuff systems performing bilateral and synchronized blood pressure recordings were used to reduce measurement variability and observer dependency [16,17]. An ABI ≤ 0.9 was considered abnormal, corresponding to a diagnosis of PAD, while an ABI > 0.9 was considered normal [4].

### 2.6. Statistical Analysis

Baseline characteristics, including age and sex, were summarized as counts and proportions by age group (<40, 40–59, 60–79, and ≥80). A contingency table was used to summarize the number of patients with abnormal, borderline, and normal ECG’s who had normal or abnormal ABIs. A chi-square test was performed to assess the association between ECG categorization and ABI classification. To evaluate whether ECG findings were associated with abnormal ABI, univariable and multivariable logistic regression models controlled for age and sex were constructed. The normal ECG group was used as the reference group for abnormal and borderline ECGs. Associations were reported as odds ratios (ORs) with 95% confidence intervals (CIs). Area under the receiver operating characteristic curve (AUROC) with 95% CI was calculated to assess the ability of ECG interpretation to predict ABI classification. Model calibration was assessed using the Hosmer–Lemeshow test. For internal validation of predictive performance, the dataset was randomly divided into 70% training and 30% test sets, with 10-fold cross-validation performed for model training. All tests were two-sided, and statistical significance was set at *p* < 0.05. Python version 3.13.3 was used for all statistical analyses [18].

## 3. Results

### 3.1. Patients

The study comprised 3381 patients across four age groups and both sexes: <40 years (females 87 [2.6%]; males 170 [5.0%]), 40–59 years (females 410 [12.1%]; males 1099 [32.5%]), 60–79 years (females 443 [13.1%]; males 1006 [29.7%]), and ≥80 years (females 47 [1.4%]; males 119 [3.5%]) (Table 1). Of these, 491 patients had both an available ECG performed within 1 year of ABI measurement and complete ECG interpretation and ABI classification data and were therefore included in the analysis.

### 3.2. ECG Categorization and ABI Classification

Among 491 patients with paired ECG and ABI data, ECG categories were distributed as abnormal (n = 345), borderline (n = 58), and normal (n = 88). The cross-tabulation with ABI status showed that for abnormal ECGs, there were 158 (45.8%) patients with an abnormal ABI. For borderline ECGs, there were 20 (34.5%) patients with an abnormal ABI. For normal ECGs, there were 25 (28.4%) patients with an abnormal ABI. A chi-square test showed a statistically significant difference in the distribution of ABI status across ECG categories (*p* = 0.0067). Specifically, patients with abnormal ECGs had the highest rate of abnormal ABI’s (45.8%). This rate drops for borderline ECGs (34.5%) and is lowest for normal ECGs (28.4%). Thus, patients with an abnormal ECG were more likely to have an abnormal ABI ≤ 0.9 (Table 2).

### 3.3. Association Between ECG Categorization and ABI Classification

The univariable logistic regression model demonstrated that patients with an abnormal ECG were more likely to have an abnormal ABI ≤ 0.9 compared to patients with a normal ECG (OR 2.25, 95% CI 1.32–3.83, *p* = 0.003). However, there was no significant difference in ABI status between patients with a borderline vs. normal ECG (OR 1.40, 95% CI 0.70–2.79, *p* = 0.340) (Table 3). After adjusting for age and sex in the multivariable logistic regression model, abnormal ECG remained independently associated with abnormal ABI (adjusted OR 2.07, 95% CI 1.24–3.46, *p* = 0.005), while there was no significant association between borderline ECG and abnormal ABI (adjusted OR 1.31, 95% CI 0.64–2.68, *p* = 0.455) (Table 4). The model predicting abnormal ABI using ECG interpretation achieved an AUROC of 0.73 (95% CI 0.68–0.78). The model demonstrated good calibration with a Hosmer–Lemeshow χ^2^ of 5.0 (*p* = 0.76).

## 4. Discussion

### 4.1. Summary of Findings

In this retrospective case–control study of patients with paired ECG and ABI data, abnormal ECG findings were significantly associated with abnormal ABI. Patients with abnormal ECGs had a higher prevalence of ABI ≤ 0.9 and more than two-fold increased odds of abnormal ABI compared to patients with normal ECGs, whereas borderline ECGs were not significantly associated with ABI status. ECG categorization demonstrated moderate discrimination and good calibration for predicting abnormal ABI. Together, these findings suggest that clinically significant ECG abnormalities may reflect underlying PAD and could help identify patients who may benefit from further vascular assessment, particularly in settings where ABI testing is less accessible.

### 4.2. Comparison to Existing Literature

Our findings align with a growing body of literature demonstrating that ECG abnormalities may reflect systemic atherosclerosis and are associated with adverse cardiovascular outcomes [6]. Prior studies have shown that a range of ECG abnormalities, including Q-wave abnormalities, ST–T wave changes, left ventricular hypertrophy, and artificial intelligence-derived ECG markers, are associated with increased risks of CAD, stroke, carotid atherosclerosis progression, cardiovascular mortality, and all-cause mortality [9,10,11,19,20,21]. Collectively, these studies suggest that ECG abnormalities may reflect cumulative cardiovascular injury and diffuse vascular disease rather than isolated cardiac pathology [9,10,11,19,20,21]. However, most prior investigations have focused on specific ECG abnormalities or cardiovascular outcomes rather than peripheral vascular disease and ABI classification [9,10,11,19,20,21]. Our study extends these findings as the first to characterize the relationship between ECG findings and ABI in patients with and without PAD. In particular, we evaluated ECG findings using a standardized, guideline-based classification framework rather than focusing on isolated abnormalities [12]. In doing so, we demonstrate that global ECG interpretation, reflecting cumulative electrical and structural cardiac abnormalities, correlates with atherosclerotic disease in the peripheral arteries [12]. These findings support the concept that ECG abnormalities are not solely indicators of cardiac pathology, but may be associated with diffuse atherosclerotic disease involving multiple vascular beds, supporting the concept and clinical importance of polyvascular disease [22].

### 4.3. Explanation of Findings

Several mechanisms may explain the observed association between abnormal ECG findings and reduced ABI. First, PAD and ECG abnormalities share common cardiovascular risk factors, including advanced age, smoking, diabetes mellitus, hypertension, and dyslipidemia [8,9,23]. These factors promote endothelial dysfunction, inflammation, and progressive atherosclerosis, leading to both CAD and PAD [24,25,26]. Second, abnormal ECG findings often reflect downstream consequences of chronic cardiovascular injury [27,28]. For example, Q waves and ST–T changes may indicate prior myocardial infarction or ongoing ischemia, while left ventricular hypertrophy may reflect long-standing pressure overload [27,28]. Conduction abnormalities may arise from structural remodeling or fibrosis of the cardiac conduction system [27,28]. Collectively, these abnormalities may serve as surrogate markers of cumulative cardiovascular burden and systemic vascular disease, including PAD [27,28]. Third, borderline ECG findings are, by definition, nonspecific and of uncertain clinical significance [12]. These findings may represent normal physiological variation or early, subclinical changes that do not yet reflect established atherosclerotic disease [12]. This likely explains the lack of a significant association between borderline ECGs and abnormal ABI observed in our analysis [12]. Thus, careful clinical interpretation of ECGs is needed to assess for the potential presence of polyvascular disease, including PAD.

### 4.4. Implications

The clinical implications of these findings are important, particularly in the context of PAD underdiagnosis [3]. ABI measurement, while non-invasive and reliable, requires dedicated equipment and trained personnel, which may limit its use in certain settings, including low-resource and/or rural areas [4]. In contrast, ECG is widely available, inexpensive, and frequently performed across a variety of healthcare settings [6]. Our results suggest that routinely available ECG data may provide adjunctive information regarding systemic vascular risk [1]. ECG should not be considered a diagnostic substitute for ABI, vascular imaging, or comprehensive clinical assessment for PAD; however, abnormal ECG findings may prompt consideration of further vascular evaluation in selected patients [1]. Importantly, findings from our study should be cautiously interpreted given its observational design, which limits causal inference. Specifically, the observed associations between ECG findings and ABI likely reflect shared cardiovascular risk factors and systemic atherosclerotic burden rather than a direct mechanistic relationship between ECG abnormalities and PAD. Importantly, because several traditional cardiovascular risk factors and comorbidities, including smoking status, dyslipidemia, cholesterol levels, hypertension, diabetes, medication use, and other cardiovascular risk factors, were not available for adjustment, residual confounding may have influenced the observed associations. Accordingly, the findings should be interpreted cautiously and considered hypothesis-generating. From a broader perspective, these findings reinforce the concept and clinical importance of systemic atherosclerosis leading to polyvascular disease and highlight the value of leveraging various sources of routinely collected clinical data to improve the detection of vascular disease across different organ systems [29,30].

The clinical implications of these findings should be interpreted cautiously. PAD diagnosis and assessment continue to rely on clinical history, physical examination, ABI testing, and vascular imaging when indicated [1]. While ECG is widely available and routinely performed across healthcare settings, ECG abnormalities are nonspecific and may reflect a broad range of underlying cardiovascular conditions rather than PAD alone [6]. Accordingly, ECG should not be considered a substitute for ABI testing or established vascular assessment strategies [1,6]. However, the observed association between abnormal ECG findings and abnormal ABI suggests that routinely collected ECG data may reflect broader systemic atherosclerotic burden and polyvascular disease [29,30]. As such, abnormal ECG findings may prompt consideration of further vascular evaluation in selected patients, particularly those with multiple cardiovascular risk factors [1]. However, the current findings are insufficient to support ECG use as a routine screening or risk-stratification tool for PAD in clinical practice. Further prospective studies are needed to determine whether ECG-derived information provides incremental value beyond standard clinical assessment for PAD and cardiovascular risk stratification [1,6].

### 4.5. Limitations

Several limitations should be considered. First, the retrospective case–control design limits causal inference regarding the temporal relationship between ECG abnormalities and PAD development. Although ECG abnormalities may evolve over time, we used the most recent ECG performed within 1 year of ABI measurement to reduce temporal variability between assessments. Nevertheless, interval changes in ECG status may have occurred and could have influenced ECG classification at the time of ABI measurement. Furthermore, the relatively small number of patients in the borderline ECG group may have limited statistical power to detect significant associations for this subgroup, and contributed to wider confidence intervals with a lack of statistical significance. Larger, prospective, and temporally controlled studies are needed to confirm and extend our findings. Second, selection bias may have been introduced by restricting the analysis to patients with available paired ECG and ABI data, which may not be representative of the broader study population. Consequently, the observed associations should be interpreted cautiously. Larger and more representative cohorts are needed to reduce the risk of selection bias. Third, residual confounding from unmeasured variables may have influenced the observed associations. Important cardiovascular risk factors and comorbidities, including smoking status, hypertension, diabetes, dyslipidemia, cholesterol levels, medication use, family history, and other markers of cardiovascular risk, were not available for inclusion in the multivariable models. Because these factors are associated with both ECG abnormalities and PAD, the observed relationship between ECG findings and ABI may partly reflect shared cardiovascular risk burden rather than an independent association. Consequently, the robustness of the findings may be reduced, and caution is warranted when interpreting the results and generalizing them to broader populations. Future prospective studies incorporating comprehensive cardiovascular risk profiling and more extensive multivariable adjustment are needed to validate these findings. Fourth, ECG interpretation was performed by a single board-certified cardiologist, and inter-observer variability was not assessed. Although standardized guideline-based reporting criteria were used and the interpreter was blinded to ABI results, the potential for ECG misclassification remains and may have influenced the observed associations. Future studies incorporating multiple independent ECG reviewers and assessment of inter-observer agreement will be important to further evaluate the reproducibility and reliability of ECG categorization in this context. Fifth, the moderate discriminatory performance observed in this study (AUROC 0.73) may limit the clinical utility of ECG categorization as a standalone tool for PAD assessment. Furthermore, ECG abnormalities are heterogeneous and often nonspecific, reflecting a broad range of underlying cardiac conditions rather than PAD alone. Future studies incorporating clinical risk factors, laboratory parameters, imaging findings, and more granular ECG features may help improve predictive performance and better define the potential role of ECG-based approaches in vascular risk assessment. Finally, this was a single-center study, which may limit the generalizability of our findings to other populations. Future multicenter studies will be important to externally validate these findings.

## 5. Conclusions

In this retrospective case–control study, abnormal ECG findings were associated with abnormal ABI and may reflect underlying systemic atherosclerotic disease. While borderline ECG abnormalities did not appear to confer increased risk, clinically significant ECG changes were associated with a higher likelihood of underlying PAD. These findings support further investigation into whether routinely collected ECG data may complement vascular risk assessment, particularly in lower-resource settings where ABI testing is not readily available. Importantly, the findings should be interpreted in the context of the observational nature of the study, which limits causal inference. Future prospective multicenter studies are warranted to validate these findings, explore causal relationships, and evaluate whether the integration of ECG-based risk assessment into clinical workflows can improve the detection and management of PAD.

## Figures and Tables

**Table 1:** Baseline characteristics of cohort.

Age Group (Years)	Females (n, %)	Males (n, %)	Total (n, %)
<40	87 (2.6%)	170 (5.0%)	257 (7.6%)
40–59	410 (12.1%)	1099 (32.5%)	1509 (44.6%)
60–79	443 (13.1%)	1006 (29.7%)	1449 (42.9%)
≥80	47 (1.4%)	119 (3.5%)	166 (4.9%)

**Table 2:** Contingency table of electrocardiogram categorization and ankle brachial index classification.

ECG Category	Normal ABI > 0.9 (n = 288)	Abnormal ABI ≤ 0.9 (n = 203)	Percentage of Patients with an Abnormal ABI
Abnormal (n = 345)	187	158	45.8%
Borderline (n = 58)	38	20	34.5%
Normal (n = 88)	63	25	28.4%

Abbreviations: ECG (electrocardiogram), ABI (ankle brachial index).

**Table 3:** Univariable logistic regression model assessing the association between electrocardiogram interpretation and abnormal ankle brachial index ≤ 0.9.

ECG Category	Odds Ratio	95% CI	*p*-Value
Abnormal vs. Normal	2.25	1.32–3.83	0.003
Borderline vs. Normal	1.40	0.70–2.79	0.340

Abbreviations: ECG (electrocardiogram) and CI (confidence interval).

**Table 4:** Multivariable logistic regression model assessing the association between electrocardiogram interpretation and abnormal ankle brachial index ≤ 0.9.

ECG Category	Adjusted Odds Ratio *	95% CI	*p*-Value
Abnormal vs. Normal	2.07	1.24–3.46	0.005
Borderline vs. Normal	1.31	0.64–2.68	0.455

* Adjusted for age and sex. Abbreviations: ECG (electrocardiogram) and CI (confidence interval).

## Data Availability

The original contributions presented in the study are included in the article; further inquiries can be directed to the corresponding author.
